# High prevalence of myopia and low hyperopia reserve in 4411 Chinese primary school students and associated risk factors

**DOI:** 10.1186/s12886-022-02436-5

**Published:** 2022-05-11

**Authors:** Yu Yue, Xianmao Liu, Shu Yi, Bo Liu, Hong Yi, Hong Li

**Affiliations:** 1 Department of Ophthalmology, Chongqing Key Laboratory of Ophthalmology, First Affiliated Hospital of Chongqing Medical University, Chongqing Eye Institute, Chongqing Branch of National Clinical Research Center for Ocular Diseases, 1 Youyi Road, Chongqing, 400016 China; 2 Department of Ophthalmology, Chongqing General Hospital, University of Chinese Academy of Sciences, 104 Pipashan Road, Chongqing, 400014 China; 3 Department of Ophthalmology, Southwest Hospital, Southwest Eye Hospital, Third Military Medical University (Army Medical University), Chongqing, 400038 China

**Keywords:** Myopia, Prevalence, Ocular biometrics, Risk factors

## Abstract

**Purpose:**

To investigate the prevalence of myopia in Chinese primary school students and their ocular biometrics including axial length (AL), corneal radius of curvature (CRC) and spherical equivalent refraction (SER). To analyze their association with potential myopia risk factors, such as body mass index (BMI), cram school, time of outdoor activity and electronic screen use.

**Methods:**

In this cross-sectional study of 4500 primary school students from 5 schools, participants underwent refraction using non-cycloplegic autorefractor and visual acuity testing. A follow-up study in the same schools was conducted in 2022. Myopia was defined as SER ≤ -0.50 diopter (D) and uncorrected visual acuity (UCVA) < 0.00 logMAR (6/6). Logistic regression models were used to determine factors associated with myopia.

**Results:**

After excluding 389 participants, the overall prevalence of myopia was 33.6%. The prevalence of high myopia was 0.6%. The prevalence of myopia in girls was significantly higher than that in boys (37.6% vs. 30.0%, *p* < 0.001). The height, weight and BMI were significantly associated with AL (*r* = 0.471, *r* = 0.440, *r* = 0.276, *p* < 0.001, respectively). AL/CRC ratio was more highly correlated with SER than AL alone. Regression analysis showed that AL/CRC and hyperopia reserve were associated with myopia onset in the subsequent year (*F* = 201.557, *p* < 0.001; *F* = 68.934, *p* < 0.001). The cut point of hyperopia reserve for myopia in the subsequent year for grade 1 students was + 0.31D. Age (*p* < 0.001), parental myopia (*p* = 0.001) and lack of outdoor activity between classes (*p* = 0.049) were independently associated with higher prevalence rates of myopia.

**Conclusion:**

The prevalence of myopia among Chinese schoolchildren is alarming high. Consistent with previous cross-sectional data, AL/CRC and hyperopia reserve could function as myopia detection indicators. The hyperopia reserve among children aged between 6 ~ 7 years was low. Healthcare providers need to raise parents’ awareness of the importance of regular eye examination and proper optical correction.

**Supplementary Information:**

The online version contains supplementary material available at 10.1186/s12886-022-02436-5.

## Introduction

Myopia, the most common refractive error, is the main cause of presenting visual impairment worldwide. The prevalence of myopia has been observed to be increasing at a dramatic rate in young East Asians. In children aged 6–8 years, 18.0% ~ 34.7% were myopic in developed areas including Beijing [1], Hongkong [2] and Singapore [3]. According to a recent meta-analysis summarizing the prevalence of myopia in 7 to 12-year-old children in China, the myopia prevalence has increased from 25.3% to 32.8% before as compared to after 2008 [4].

Although the refractive error can be corrected optically, myopia has been associated with complications such as retinal detachment, myopic macular degeneration and choroidal neovascularization [5, 6]. These complications can cause irreversible visual impairment and considerable visual morbidity. Among Chinese university students, studies reported that 86.8% had myopia in Nanjing [7], 94.9% had myopia and 19.5% had high myopia in Shanghai [8]. The high prevalence of myopia and high myopia in Chinese students has strong impact on their quality of life and inevitably increases the socioeconomic burden. Therefore, it is of prime importance to identify primary school students at a higher risk of pathological myopia development in order to start close ophthalmic surveillance and control myopia progression with appropriate measures.

Currently, researchers often evaluate the refractive status in large cohorts of school children by refraction without cycloplegia, and by measuring axial length (AL), corneal radius of curvature (CRC) and spherical equivalent refraction (SER). However, assessing the likelihood of future myopia development is still difficult. Scheiman et al. reported that the ratio of AL to CRC(AL/CRC) could be a better marker of myopia progression than AL alone [9]. Hyperopia reserve is defined as the physiological hyperopia during infancy and young childhood, which will decrease during emmetropization [10]. It is estimated that the lack of hyperopia reserve might be a predictor of future myopia formation [11].

The mechanism of myopia formation and progression still remains uncertain. Genetic contribution to refractive error is important nevertheless it could not explain the recent considerable increase in myopia prevalence. Based on modern epidemiological and genetic analysis, it is generally agreed that the formation of school myopia is a complex developmental process susceptible to environmental modulation [12]. Environmental factors such as educational pressures and reductions of time outdoors were causal factors of myopia [13]. Nowadays, the electronic screen use [14], the body mass index (BMI) were also reported to be potential myopia risk factors [15]. Hence finding the major causal factors and ameliorating children’s studying condition and daily behavior could be the key to myopia control.

In this study, we aimed to determine the prevalence of myopia in primary school students in Chongqing, the largest city in China. And we aimed to analyze the refractive characteristics and their association with potential myopia risk factors, such as BMI, time of cram school, electronic screen use, etc. We also conducted the evaluation of the ratio of AL to CRC, the hyperopia reserve as potential marker or predictor of myopia formation in this population.

## Methods

### Study design and population

In January of 2021 and 2022, we conducted a survey of myopia prevalence in children from 5 primary schools in Chongqing, China. Three schools are located in urban area and two schools are from less developed towns. Every student from each grade of the 5 primary schools was invited in this survey. The purpose and procedures of the study were explained to all of them. A written consent form was signed by the parents. All of the study protocols conformed to the Tenets of the Declaration of Helsinki and the research protocols were approved by the Committee of Ethics in Chongqing People’s Hospital. Ethical approval number: KYS2021-011–01. Students who had systemic pathologies were excluded.

### Ocular examination

Visual acuity was measured as uncorrected visual acuity (UCVA) (using a logarithmic VA chart) at a distance of 5 m. Refraction was measured three times in each eye in a darkened room without cycloplegia with the autorefractometer KR-800 (Topcon, Japan). Axial length, CRC and central corneal thickness were measured with the AL-Scan (NIDEK Ltd., Tokyo, Japan). Axial length was defined as the distance between the tear film and the retinal pigment epithelium. All the data were uploaded in an online database right away. Myopia was defined as a SER ≤ -0.50 diopters (D) and UCVA < 0.00 logMAR (6/6) in either eye. High myopia was defined as a SER ≤ -6.00 D. Hyperopia was categorized by the degree of refractive error. Low hyperopia was defined as a SER ≤  + 2.00 D. Moderate to high hyperopia was defined as a SER ≥  + 2.25 D. After excluding subjects with moderate to high hyperopia, an adequate hyperopia reserve was defined as SER ≥  + 1.00 D for 6–8 years old, ≥  + 0.75D for 9 years old, ≥  + 0.50 D for 10 years old, ≥  + 0.25 D for 11 years old and ≥ 0 D for 12 years old according to *the handbook for prevention and control of myopia in children and adolescents*by the National Health Commission of the People’s Republic of China [16].

As for quality control, 5% of participants were randomly chosen from the database at the end of day during the fieldwork. The UCVA and auto-refraction were retested. Data with an error rate lower than 5% was accepted as valid.

### BMI measurements

Height and weight were measured at the same time by SH-200 height-weight measurement body scale meter (Shanghe Ltd., Zhengzhou, China) with the subject standing barefoot on the base, without shoes and heavy coats. BMI was calculated as weight/height and recorded in kilograms per square meter (kg/m^2^).

### Questionnaire

A questionnaire was developed by two ophthalmologists from Department of Ophthalmology, Chongqing General Hospital and answered by the parents of our subjects. The questionnaire included questions about the educational attainment of parents, parental myopia, electronic screen use, cram school, reading posture and duration of outdoor activities (See Supplementary Material 1 and 2).

### Statistical analysis

All statistical analyses were performed with the SPSS statistics 20 for Windows (SPSS Inc., IBM, Somers, New York, USA). AL, SER, ACD, CRC and AL/CRC were not distributed normally based on Shapiro–Wilk test. Thus, the significance of the biometric differences between different grades (6 grades in total) were calculated by Kruskal–Wallis test. The difference of myopia prevalence between different genders were calculated by Chi square test. Linear regression analysis was conducted to evaluate the AL/CRC and hyperopia reserve as potential predictors of myopia onset in 2022. The cut points of AL/CRC and SER to predict the onset of myopia in subsequent grades were determined using the Youden index, the best combination of sensitivity and specificity. The association between height, weight and BMI and AL were analyzed by Spearman’s correlation analyses. Univariate analysis was performed to evaluate potential associations. These included age, sex, BMI, parental myopia, outdoor activity during break between classes, time of PE lessons, time of cram school, electronic screen use. Afterwards, multivariate logistic regression modeling was performed to analyze all statistically significant factors found in the univariate analysis. *P* values are 2 sided and *P* < 0.05 was considered statistically significant.

## Results

### Demographic characteristics

Four thousand five hundred participants were recruited in the initial research. A total of 4411 participants were available for the statistical analysis after excluding 88 subjects with missing data, 1 subject diagnosed with Down syndrome. Table 1 presents the demographic characteristics of the analysis cohort by myopic or non-myopic. The response rate of the questionnaire was 749 out of 4411. One hundred and thirty-two subjects chose “unknown” in the questionnaire and were excluded during analysis.

**Table 1:** Demographic characteristics

**Variables**	Myopic	Non-myopic	Total	*P* value
**Age (years)**	10 (8, 11)	8 (7, 10)	9 (7, 10)	< 0.001^a^
**Height (cm)**	141.0 (133.0, 149.0)	132.0 (125.0, 141.0)	135.0 (127.0, 144.5)	< 0.001^a^
**Weight (kg)**	36.1 (29.7, 44.4)	29.9 (25.3, 37.0)	31.8 (26.4, 40.0)	< 0.001^a^
**BMI (kg/m**^**2**^**)**	17.95 (16.43, 20.36)	17.07 (15.81, 18.94)	17.36 (15.99, 19.46)	< 0.001^a^
**Gender**				< 0.001^b^
Male	684 (30.0%)	1598 (70.0%)	2282	
female	800 (37.6%)	1329 (62.4%)	2129	
**Wearing spectacles**				< 0.001^b^
Yes	377 (80.9%)	89 (19.1%)	466	
No	1107 (28.1%)	2838 (71.9%)	3945	
**Total**	1484 (33.6%)	2927 (66.4%)	4411	

^a^Mann-Whitney U test

^b^Chi-square test

Data are presented as medians and quartiles (p25, p75) or as n (%)

### Prevalence of myopia

**Fig. 1:**
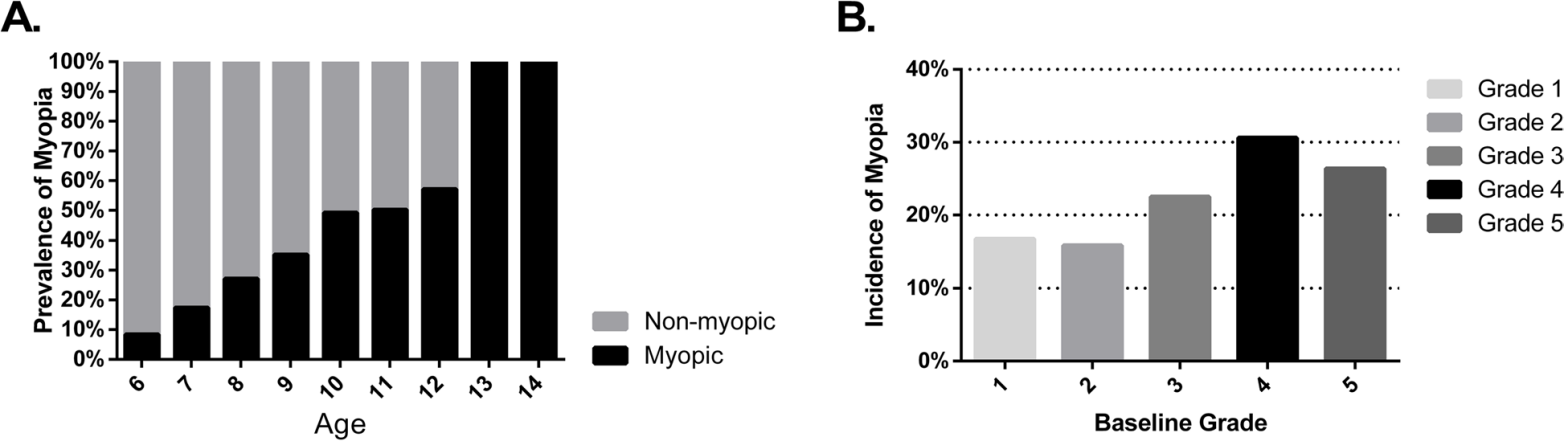
**A** Prevalence rate of myopia based on the age of students; (**B**) Incidence rate of myopia in each grade in 2022

The overall prevalence of myopia was 33.6% (1484 of 4411). The prevalence of high myopia was 0.6% (27 of 4411). The prevalence rate of myopia in different grades and sexes was shown in Table 2. Figure 1 (A) presented the prevalence rate of myopia based on their age. The prevalence of myopia increased significantly as the grade levels rose (x^2^ = 522.169, *p* < 0.001), from 8.6% in first grade to 55.6% in sixth grade. After adjusted by Bonferroni method, significant difference was found between every two grades from 1^st^ to 4^th^ grade, and between 4 and 6^th^ grade. There was no significant difference in the prevalence of myopia between 4 and 5^th^ grade, 5^th^ and 6^th^ grade. Figure 1 (B) demonstrated the incidence rate of myopia in each grade. The highest incidence, 30.6%, was in the 4^th^ grade. The prevalence of myopia in girls was significantly higher than that in boys (37.6% vs. 30.0%, x^2^ = 28.517, *p* < 0.001).

**Table 2:** Prevalence of myopia in different grades and sexes

**Grade**	**Male**		**Female**		**Total**		**Increase rate between grades** **%**
	**Myopic**	**%**	**Myopic**	**%**		%	
1^st^	42	5.1	28	3.4	817	8.6	
2^nd^	78	9.6	96	11.8	812	21.4	12.9^a^
3^rd^	141	17.2	127	15.5	819	32.7	11.3^a^
4^th^	137	19.4	180	25.5	705	45.0	12.2^a^
5^th^	148	21.2	196	28.0	699	49.2	4.2
6^th^	138	24.7	173	30.9	559	55.6	6.4
Total	684	15.5	800	18.1	4411	33.6	

^a^statistically significant

### Refraction and ocular biometric parameters

**Fig. 2:**
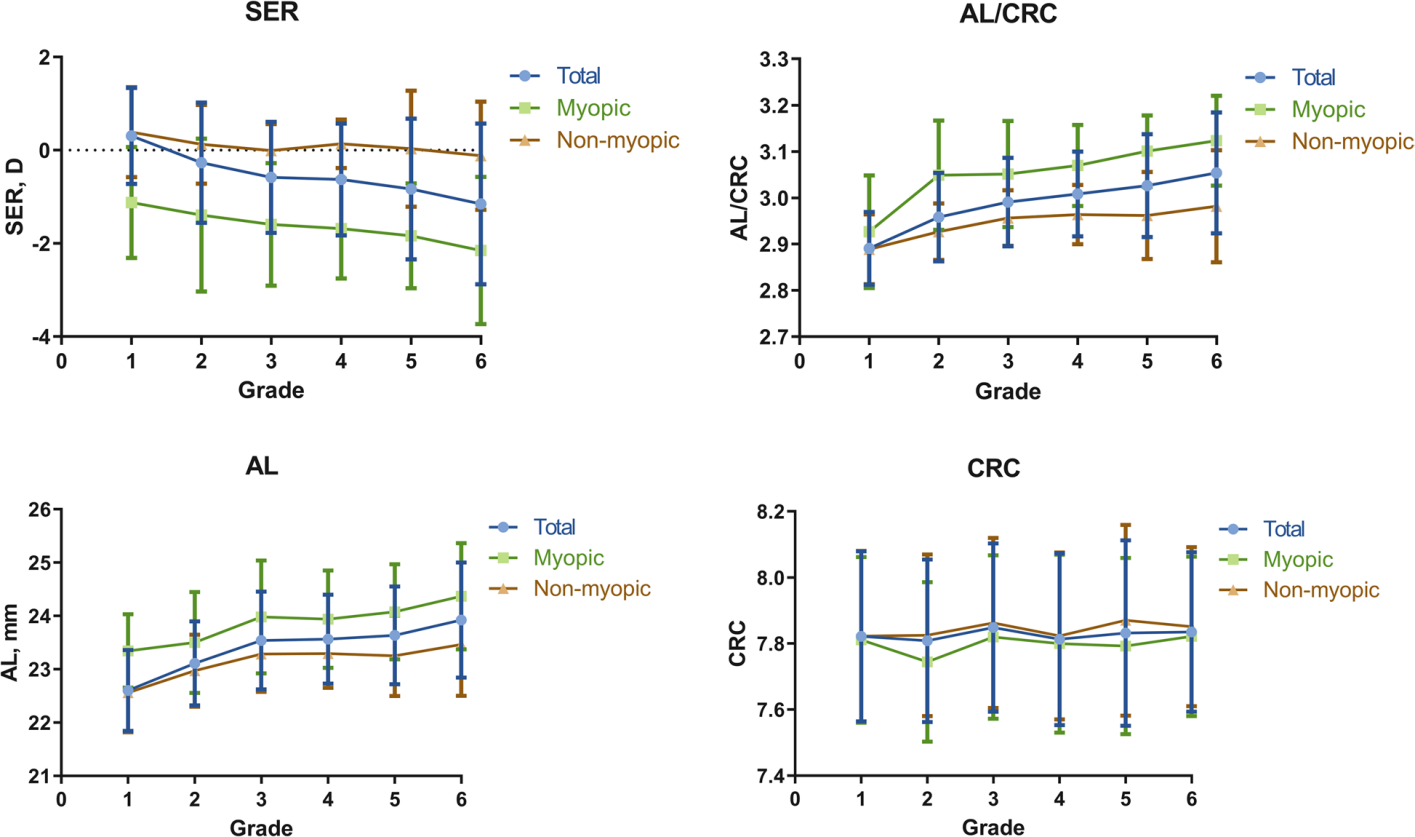
Dots represents means and bars represents SDs. SER: spherical equivalent refraction; AL: axial length; CRC: corneal radius of curvature; AL/CRC: ratio of AL to CRC

The mean SERs were 0.31 D, -0.27 D, -0.58 D, -0.63D, -0.83D and -1.15D for participants from 1^st^ to 6^th^ grade respectively (Fig. 2). Significant differences (p < 0.01) were found between each two grades, except between 4 and 5^th^ grade (*p* = 0.117). Spearman’s correlation test showed that the AL and AL/CRC were closely associated with SER(AL: *r* = -0.531, *p* < 0.001; AL/CRC: *r* = -0.639, *p* < 0.001).

The mean ALs were 22.60, 23.11, 23.54, 23.56, 23.63 and 23.92 mm from 1^st^ to 6^th^ grade respectively. Significant differences (*p* < 0.01) were found between each two grades, except between 3^rd^ and 4^th^ (*p* = 0.060), 5^th^ and 6^th^ grade (*p* = 0.268). The mean ratio of AL to CRC were 2.89, 2.96, 2.99, 3.01, 3.03, 3.05 from 1^st^ to 6^th^ grade respectively. Significant differences (*p* < 0.01) were found between each two grades, except between 5 and 6^th^ grade (*p* = 0.931). The refractive characteristics of the myopic and non-myopic groups were shown in Table 3. Univariate regression analysis demonstrated that AL/CRC was associated with myopia onset in the subsequent year (*F* = 201.557, *p* < 0.001).

**Table 3:** Refractive characteristics

**Variables**	Myopic	Non-myopic	Total	*P* value (Myopic vs. Non-myopic)
AL (mm)	23.92 (23.35, 24.61)	23.01 (22.50, 23.49)	23.27 (22.69, 23.91)	< 0.001^a^
CRC (mm)	7.79 (7.62, 7.96)	7.84 (7.67, 8.00)	7.82 (7.65, 7.99)	0.03 ^a^
SER (D)	-1.50 (-2.50, -0.88)	+ 0.13 (-0.25, + 0.50)	-0.13 (-1.13, -0.13)	< 0.001 ^a^
ACD (mm)	3.77 (3.63, 3.93)	3.56 (3.39, 3.74)	3.64 (3.45, 3.82)	< 0.001 ^a^
CCT(μm)	548.97 ± 32.67	550.75 ± 31.22	550.14 ± 31.72	0.357 ^b^
AL/CRC	3.07(3.01, 3.14)	2.94(2.89, 2.98)	2.97(2.91, 3.04)	< 0.001 ^a^

Data are presented as medians and quartiles (p25, p75) or as the means with SD

^a^Mann-Whitney U test

^b^Independent t-test

**Fig. 3:**
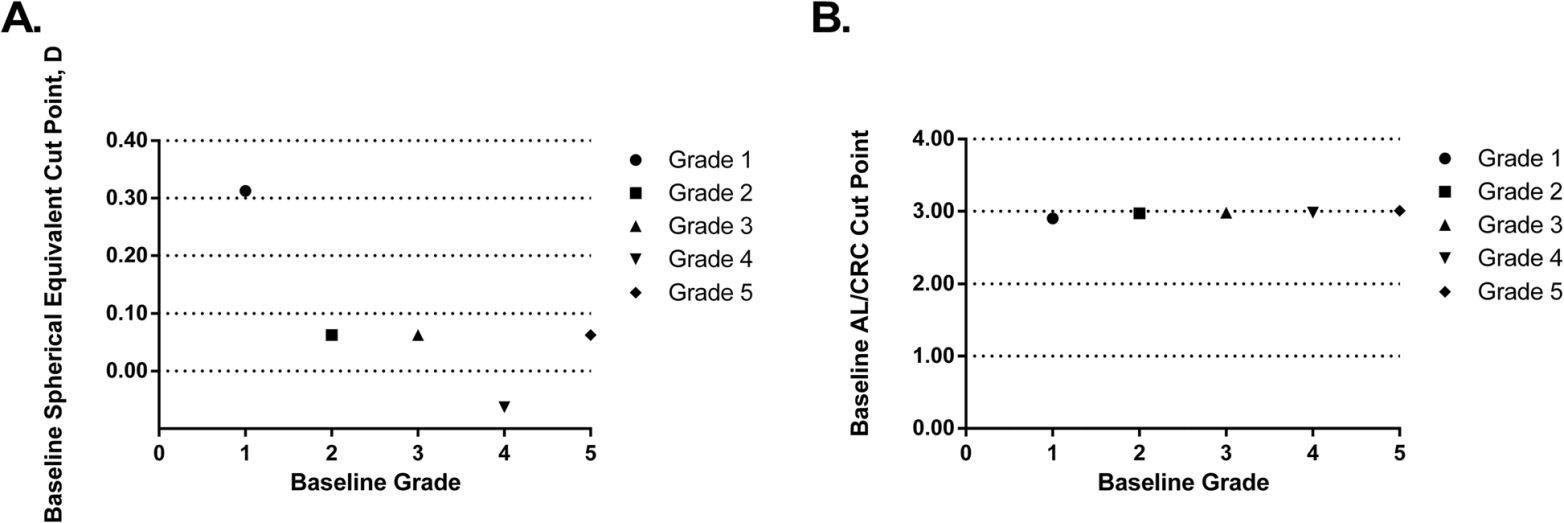
Cut Points for Myopia Onset in the Subsequent Year: **A** the baseline spherical equivalent; (**B**) the baseline AL/CRC: ratio of AL to CRC

There were 384 participants (8.7%) out of 4411 with adequate hyperopia reserve in total according to the standard set by the *handbook for prevention and control of myopia in children and adolescents* by the National Health Commission of the People’s Republic of China. The prevalence of moderate and hyperopia was 0.8% (36/4411). The percentage of subjects with adequate hyperopia reserve was low, especially in younger participants (4.4 ~ 6.1% for children aged ≤ 8 vs. 6.2 ~ 10.7% for children aged between 9 to 10). Univariate regression analysis showed that hyperopia reserve was associated with myopia onset in the subsequent year (*F* = 68.934, *p* < 0.001) in a statistically significant way. The cut points of SER and AL/CRC for myopia onset in 2022 were presented in Fig. 3. The cut point of SER for myopia in the subsequent year for grade 1 students was + 0.31D. And the cut point of AL/CRC for grade 1 students was 2.90.

### Relationship between body stature and refractive characteristics

The height, weight and BMI were found to be significantly associated with AL (*r* = 0.471, *p* < 0.001; *r* = 0.440, *p* < 0.001; *r* = 0.276, *p* < 0.001, respectively), SER (*r* = -0.376, *p* < 0.001; *r* = -0.334, *p* < 0.001; *r* = -0.189, *p* < 0.001, respectively) and AL/CRC (*r* = 0.445, *p* < 0.001; *r* = 0.406, *p* < 0.001; *r* = 0.240, *p* < 0.001, respectively).

### Multiple logistic regression analysis

Univariate analysis showed that educational attainment of parents, time of homework, near visual work, reading and writing posture, time of outdoor activities were not related with incidence of myopia (results shown in Supplementary Material 3). Relevant risk factors validated by univariate analysis were listed in Table 4. Multiple logistic regression analysis was then performed. Results showed that parental myopia was associated with a greater prevalence of myopia (Table 4).

**Table 4:** Analysis of risk factors of myopia by multiple logistic regression

	N (%)	Risk of Myopia	
		OR (95%CI)	p
**Age**^**a**^	617	1.66 (1.48–1.86)	< 0.001^a^
**BMI**	617		0.920
**Female**	294 (47.6%)		0.395
**Parent myopia**^**a**^			
Non	302 (48.9%)	ref	
One myopic	220 (35.7%)	1.98 (1.32–2.97)	0.001^a^
both	95 (15.4%)	2.28 (1.36–3.83)	0.002^a^
**Take a break outdoors**^**a**^			
No	414 (67.1%)	ref	
Yes	203 (32.9%)	0.67 (0.45–1.00)	0.049^a^
**PE class**			0.675
< 3	66 (10.7%)		0.725
3	306 (49.6%)		0.974
4	195 (31.6%)		0.621
5	53 (8.6%)		0.585
> 5	26 (4.2%)		0.939
**Cram school**			0.384
No	253 (41.0%)		0.481
< 1 h	56 (9.1%)		0.734
1-2 h	135 (21.9%)		0.273
2-3 h	73 (11.8%)		0.961
> 3 h	129 (20.9%)		0.434
**Electronic screen use**			0.445
No	40 (6.5%)		0.363
< 30 min	203 (32.9%)		0.638
30-60 min	189 (30.6%)		0.668
1-2 h	118 (19.1%)		0.390
2-3 h	38 (6.2%)		0.094
> 3 h	58 (9.4%)		0.492

Data are presented as n (%)

^a^Statistically significant

Other independently associated factors for myopia prevalence were age (*p* < 0.001) and lack of outdoor activity during break between classes (*p* = 0.049). On the contrary, electronic screen use, time of cram school and time of PE class were not associated with myopia prevalence in the multiple logistic regression. Parental myopia was also associated with longer axial length (regression coefficient 0.100 [95% CI, 0.026–0.174], *p* = 0.008) and greater AL/CRC (regression coefficient 0.012[95% CI, 0.004–0.020], *p* = 0.005) (Table 5).

**Table 5:** Analysis of association between risk factors and AL and AL/CRC by multiple linear regression

	AL		AL/CRC	
	regression coefficient, 95% CI	*p* value	regression coefficient, 95% CI	*p* value
**Age**^**a**^	0.266 [0.224–0.308]	< 0.001^a^	0.029[0.025–0.034]	< 0.001^a^
**BMI**^**a**^	0.024 [0.001–0.047]	0.044^a^	0.002[0.000–0.005]	0.102
**Female**^**a**^	0.582 [0.453–0.710]	< 0.001^a^	0.021[0.007–0.036]	0.004^a^
**Parental myopia**^**a**^	0.100 [0.026–0.174]	0.008^a^	0.012[0.004–0.020]	0.005^a^
**Take a break outdoors**	-0.090 [-0.225–0.046]	0.194	-0.008[-0.023–0.008]	0.318
**PE class**	0.016 [-0.054–0.085]	0.662	0.005[-0.003–0.013]	0.229
**Cram school**^**a**^	0.045 [0.002–0.089]	0.042^a^	0.005[0.000–0.010]	0.047^a^
**Electronic screen use**	-0.010 [-0.060–0.039]	0.682	0.000[-0.006–0.006]	0.985

^a^Statistically significant

Multiple logistic regression analysis showed that older age, the female sex, higher BMI, more cram school classes were independently associated with longer axial length. Factors which independently associated with greater AL/CRC were similar, except BMI.

## Discussion

In this study, we investigated the prevalence of myopia, the refractive characteristics and their associated risk factors among primary school children in Chongqing. The overall prevalence of myopia among primary school children in Chongqing was 33.6%. The prevalence of high myopia was 0.6%. The prevalence of myopia rose dramatically from 8.6% in the 1^st^ grade to 55.6% in the 6^th^ grade. Compared with boys, more girls were myopic (37.6% vs. 30.0%). A high correlation between AL/CRC and SER was found, and its coefficient of correlation was higher than that between AL and SER (*r* = -0.639 for AL/CRC vs. *r* = -0.531 for AL). We investigated the hyperopia reserve among our subjects and found only 8.7% with adequate hyperopia reserve. Age, lack of outdoor activity during break between classes and parental myopia were identified as independent risk factors of myopia.

The prevalence of myopia among Chinese schoolchildren is often reported to be higher than children from other ethnic groups. The myopic rate was 1.4% in South America [17] (children aged 5 ~ 15 years) and 19.6% in France [18] (children aged 0 ~ 9 years), Gryzbowski et al. summarized that the highest prevalence of myopia in children was in urban areas of China, Singapore and South Korea [19]. The prevalence of myopia in Chongqing is growing rapidly over the recent 10 years. Pi et al. reported in 2010 the prevalence of myopia was 0.42% ~ 19.34% (children aged 6 ~ 12 years) in suburban area of Chongqing [20]. While according to Xie et al., the prevalence of myopia rose to 9.9% ~ 48.8% (children aged 7 ~ 12 years) in 2020 [21]. Nevertheless, according to our study, myopic students who did not wear glasses accounted for 74.25% of the whole myopic population. Without adequate correction of refractive error, those students are at risk of having more challenges in their academic life and a more rapid progression of myopia. Those evidences showed that myopia in schoolchildren have become a major health concern in China. More measures should be taken to slow the progression of myopia and raise the rate of correction in myopic students.

Our study found that AL/CRC ratio was more highly correlated than AL alone with SER (AL: *r* = -0.531 vs. AL/CRC: *r* = -0.639). Although greater AL was associated with a greater likelihood of myopia, myopic refractions were also found in shorter eyes. The finding of this research is consistent with other population-based studies of ocular biometry in children. Ip et al. reported that in Australian population, AL/CRC ratio correlated better with refraction than AL alone [22]. Scheiman et al. stated a larger correlation between AL/CRC and SER as myopia progressed in a longitudinal study in the United States [9]. He et al. stated that AL/CRC ratio could explain 65.7% of the variance in SER [23]. Previous studies suggested that AL/CRC could function as myopia detection indicator [24] and considered an AL/CRC ratio equal to or higher than 3 to be indicative of myopia [23, 25, 26]. The current study found that the average AL/CRC among myopic subjects was 3.08, and that among non-myopic subjects was 2.94 ± 0.08, similar to the findings from previous researches [26].

Hyperopia reserve is also a potential indicator of myopia. At birth neonates display a wide range of refractions. There is a progressive shift in mean refraction from + 2D to approximately + 0.75D. Then emmetropization continues at a slower rate after 6 years of age. Studies reported that the mean refraction is hyperopic in children at age of 6 ~ 7 years from Australia and European countries [27], while it is the lowest in Japan [28]. And Japan displays much higher rates of myopia in older children. In our study, the percentage of children with adequate hyperopia reserve was only 6.10% at age of 6 ~ 7 years. We estimate that their incidence of myopia could be greater than their seniors. However, follow-up studies are required to validate its predictability. Zadnik et al. conducted an observational cohort study in school-aged children in the United States and found that children in grade 1 with less than + 0.75D of hyperopia are at increased risk for developing myopia [11]. In our study, the cut point for myopia in the subsequent year for grade 1 students was + 0.31D, which is less hyperopic than the report by Zadnik et al. The difference could be contributed by the examination protocol without cycloplegia in our study. Clinicians and parents should pay attention to the children with less hyperopia reserve and begin to take measures in myopia prevention and control.

Our results highlight that the significant association between the height, weight and BMI with AL, SER and the AL/CRC ratio. However, after adjusted by sex, age, parental myopia, and other risk factors, BMI could be associated with longer AL (*p* = 0.044). But BMI did not raise the risk of myopia. The current results are consistent with Ye et al., [15] who demonstrated that among 482 Chinese children aged between 6 ~ 15 years, taller individuals were associated with longer ALs (*b* =  + 0.25, *p* < 0.01), deeper VCDs (*b* =  + 0.23, *p* < 0.01), higher AL/CC ratios (*b* =  + 0.04, *p* < 0.01) and more negative refractions (*b* =  − 0.48, *p*< 0.01). Nonetheless, BMI was not correlated with refraction in their multiple linear regression models. A research from Singapore analyzing the relationship between BMI and myopia presented showed that BMI is associated with longer axial length in girls but not in boys [29]. The relationship between BMI and refraction is not conclusive. An epidemiological study of myopia in Korea reported no relationship between body stature and myopia [30].

Our observation of a strong association between parental myopia and myopia in children is consistent with previous findings. The risk of developing myopia was higher when both parents had myopia than only one parent. Jiang et al. suggested that parental myopia might contribute to a child’s myopia by setting up a more myopic baseline at school age [31]. The debate over the relative contribution of genetic vs environmental influences on myopia always exists. However, the importance of both is becoming more recognized.

Our study evaluated potential risk factors such as near visual work, electronic screen use, cram schools, reading posture and duration of outdoor activities. And we found that time of homework, near visual work, reading and writing posture, time of outdoor activities were not related with incidence of myopia. In previous studies, the impact of near visual work and time of homework on myopia formation was not consistent. According to the systematic review by Huang et al., longitudinal studies of incident myopia conducted in Australia, Taiwan, Singapore, and the United States reported that near work was not a significant risk factor for myopia development [32]. Outdoor time was a significant protective factor for myopia in clinical trials and in cross-sectional studies [33]. It is inconsistent with the current study, which might be owing to the rather low response rate of questionnaires. Cram schools are prevalent in China because of the heavy educational pressure. Many parents are keen on enhancement of children’s academic performance. Time for outdoor activities is taken up by extra homework and examinations. Ku et al. reported that cram school attendance for more than 2 h/day may increase the risk of incident myopia in children from Taiwan [34]. Our study found that time of cram schools are independently associated with increase of AL and AL/CRC. Electronic screen time was shown to be related with myopia in the univariate analysis. However, in the multiple logistic regression, it was not associated with myopia prevalence. According to the systematic analysis by Wang et al., the risk of smartphone overuse on myopia was not statistically significant (OR = 1.05, 91%CI 0.98–1.13) [14]. Whether electronic screen overuse would lead to a higher risk of myopia is still debatable.

The strength of our study includes the large sample size with representative ocular biometric characteristics of schoolchildren in all grades. We demonstrated the importance of AL/CRC ratio as indicator of myopia. And we reported that hyperopia reserve could be a potential predictor of myopia in younger schoolchildren. The limitations of this study should also be discussed. We used social media mobile phone application as the platform to deliver and collect our questionnaire. The response rate is rather low, which limited our sample size when analyzing the risk factors of myopia. It might also cause a bias in our study because the parents who answered the questionnaire were possibly more attentive to the health care of their children.

## Conclusions

This study found that in primary schools in Chongqing, the prevalence of myopia is alarming high. The rate of myopic students wearing spectacles is far from satisfying. Although myopia is still uncommon in children aged between 6 ~ 7 years, the hyperopia reserve among them is low and they might be at a greater risk of developing myopia in the next few years. AL/CRC and hyperopia reserve could function as myopia detection indicators to help clinicians to identify children at greater risk of myopia development. Outdoor activity is a protective factor against myopia. Health care providers need to raise parents’ awareness of the importance of regular eye examination and proper optical correction. Children should be encouraged to walk out of their classroom during breaks between classes. Future longitudinal studies in the same population are planned to evaluate the predicative value of hyperopia reserve and other potential factors.

## Supplementary Information

**Additional file 1: Supplementary Material 1 (S1). **The questionnaire for myopia and influencing factors in English.

**Additional file 2: Supplementary Material 2 (S2).** The questionnaire for myopia and influencing factors in Chinese.

**Additional file 3: Supplementary Material 3 (S3).** Results of univariate analysis of the questionnaire for myopia and Influencing factors in Schoolchildren.

## Data Availability

The data have not been placed in any online data storage. The datasets generated and analyzed during the study are available upon request from the first author.
