# Supplementary material for: High prevalence of myopia and low hyperopia reserve in 4411 Chinese primary school students and associated risk factors
Source: BMC Ophthalmol. 2022 May 11;22:212. doi: 10.1186/s12886-022-02436-5 (PMC9092685; doi:10.1186/s12886-022-02436-5)
Supplement: Supplementary file 1 — Additional file 1: Supplementary Material 1 (S1). The questionnaire for myopia and influencing factors in English. [file 12886_2022_2436_MOESM1_ESM.doc]

Questionnaire for Myopia and Influencing factors in Schoolchildren

A、Basic Information

|  | Questions | Options |
| --- | --- | --- |
| A01 | The educational attainment of your child's father | 1. High School or Technical Secondary School 2. Junior College 3. Bachelor 4. Postgraduate |
| A02 | The educational attainment of your child's mother | 1. High School or Technical Secondary School 2. Junior College 3. Bachelor 4. Postgraduate |
| A03 | Your child's weight at birth | 1. ≧2.5 Kg 2.<2.5 Kg |
| A04 | The way your child was born | 1. Natural delivery (natural birth) 2. Caesarean section 3. Unclear |
| A05 | Whether your child is premature at birth (<37 weeks of gestation) | 1. Yes 2. No 3. Don't know |

B、Activities at School

|  | Questions | Options |
| --- | --- | --- |
| B01 | Were the desk and chair height adjusted according to your child's height? | 1. Never or desks and chairs not adjustable 2. Once in a school year 3. Once in a semester 4. Once in two to three months |
| B02 | During the break between classes, does your child usually take outdoor activities? | 1.No 2.Yes |
| B03 | How many classes' recess in a day does your child have ten minutes to walk out of the classroom to go outdoors? | 1. Every class (go to B05) 2. Two classes once 3. Basically not 4. Never |
| B04 | What is the main reason why he or she can't go outdoors for ten minutes between classes? | 1. The teacher didn't allow to go out 2. The teacher overran the classes. 3. He or she didn’t want to go out |
| B05 | How many PE classes does your child have in a week? | 1.<3 classes,2.3 classes,3.4 classes,4.5 classes，5.>5 classes 6. Don't know |
| B06 | Where does PE class generally take place? | 1. Indoors 2. Outdoors, such as on the playground, etc. |

C、Activities after school

|  | Questions | Options |
| --- | --- | --- |
| C01 | In the past week, how long did your child do homework or read and write on average every day after school? | 1. <1h, 2.1h-2h, 3.2h-3h, 4.>3h, 5. Don't know 6.No homework |
| C02 | In the past week, how long did your child take cram school classes such as English, math, and writing? | 1. <1h, 2.1h-2h, 3.2h-3h, 4.>3h, 5. Don't know 6.No cram school classes(go to C06） |
| C03 | Where did cram school classes take place? | 1. All online 2. All in classrooms 3. Both online and in classrooms, mainly online 4. Both online and in classrooms, mainly in classrooms |
| C04 | At what age does your child start to take cram school classes? | 1.Before 3 years old, 2.at 3 , 3.at 4, 4.at 5, 5.After 6 years old |
| C05 | To give your children more time to do homework or go to cram school, will you reduce the time your child spends on exercise? | 1. Often 2. Sometimes 3. No |
| C06 | Do you restrict your children's time to watch TV, play computer or video games? | 1.Yes 2.No |

D、Reading and writing posture

|  | Questions | Options |
| --- | --- | --- |
| D01 | When your child reads and writes, is his or her chest at proper distance (5-7cm) between the edge of the table? | 1. Never 2. Occasionally 3. Often 4. Always |
| D02 | Are your child’s eyes approximately 33 cm away from the book when reading and writing? | 1. Never 2. Occasionally 3. Often 4. Always |
| D03 | Does your child hold the pen about 3.3 cm away from the tip when reading and writing? | 1. Never 2. Occasionally 3. Often 4. Always |
| D04 | Does the teacher or parent remind your child to pay attention to the reading and writing posture? | 1. Never 2. Occasionally 3. Often 4. Always |

E、Electronic Screen Use

|  | Questions | Options |
| --- | --- | --- |
| E01 | In the past week, how long did your child use electronic products (including TVs, computers, mobile phones, ipads, etc.) each day for learning or other purposes on average? | 1. Never 2.< 30 min 3.30-60min 4.1-2h 5.2-3h 6.>3h |
| E02 | The type of multimedia used by the teacher in your child's class is (multiple choices available) | 1. Electronic whiteboard 2. Projection screen 3. Touch screen TV 4. No multimedia 5. Don’t know |
| E03 | What is the average time that your child’s class teacher uses multimedia devices in each lesson? | 1.Never 2.<15min 3.15-30min 4.>30min |

F、Outdoor activities

|  | Questions | Options |
| --- | --- | --- |
| F01 | In the past week, the average time your child was exposed to natural light after school every day | 1.<0.5h 2.0.5-1h 3.>1h |
| F02 | In the past week, how long did your child spend outdoors during the day on average? | 1.<1h 2.1-2h 3.2-3h 4.>3h |
| F03 | Are you and your child aware that outdoor activities have a good effect on myopia prevention and control? | 1. No 2. Not so impressed about it 3. Yes |
| F04 | If an eye expert tells you that doing homework and reading outdoors (such as in the garden, on the balcony) is of great significance for preventing myopia, what would you do? | 1. I will consider it in the future. 2. It cannot be implemented. 3. I disagree. |

G、Family History of Myopia

|  | Questions | Options |
| --- | --- | --- |
| G01 | Do the child's parents have myopia? | 1. No 2. One side has myopia. 3. Both sides have myopia. 4. Don't know |
| G02 | Do the child's parents have high myopia? | 1. No 2. One side has high myopia. 3. Both sides have high myopia. 4. Don't know |
| G03 | Do the child's maternal grandparents have myopia? | 1. No 2. One side has myopia. 3. Both sides have myopia. 4. Don't know |
| G04 | Do the child's maternal grandparents have high myopia? | 1. No 2. One side has high myopia. 3. Both sides have high myopia. 4. Don't know |
| G05 | Do the child's paternal grandparents have myopia? | 1. No 2. One side has myopia. 3. Both sides have myopia. 4. Don't know |
| G06 | Do the child's paternal grandparents have high myopia? | 1. No 2. One side has high myopia. 3. Both sides have high myopia. 4. Don't know |
