# Supplementary material for: High prevalence of myopia and low hyperopia reserve in 4411 Chinese primary school students and associated risk factors
Source: BMC Ophthalmol. 2022 May 11;22:212. doi: 10.1186/s12886-022-02436-5 (PMC9092685; doi:10.1186/s12886-022-02436-5)
Supplement: Supplementary file 2 — Additional file 2: Supplementary Material 2 (S2). The questionnaire for myopia and influencing factors in Chinese. [file 12886_2022_2436_MOESM2_ESM.doc]

学生视力不良及影响因素专项调查表

A、基本信息

|  | 调查内容 | 选项 |
| --- | --- | --- |
| A01 | 您的孩子父亲的教育程度 | 1.高中或中专2.大专3.本科4.研究生 |
| A02 | 您的孩子母亲的教育程度 | 1.高中或中专2.大专3.本科4.研究生 |
| A03 | 您的孩子出生时的体重 | 1.大于5斤 2.小于5斤 |
| A04 | 您的孩子的出生方式 | 1.自然分娩（顺产）2.剖腹产（剖宫产）3.不清楚 |
| A05 | 您的孩子出生时是否早产（孕周 <37周） | 1.是2.否3.不知道 |

B、学生校内用眼环境

|  | 调查内容 | 选项 |
| --- | --- | --- |
| B01 | 课桌椅高度会根据您的孩子的身高进行调整吗？ | 1.从不或课桌椅不可调 2.一学年一次 3.一学期一次4.两至三个月一次 |
| B02 | 在课间休息时，您的孩子一般在哪里活动？ | 1.教学楼内 2.户外（如操场等） |
| B03 | 您的孩子一天中有几节课的课间十分钟可以走出教室去户外？ | 1.每节课都能(跳到B05)2.两节课一次3.基本不能 4.从来不能 |
| B04 | 课间十分钟不能走出教室去户外的主要原因是？ | 1.老师不让出去2.老师拖堂，没时间出去3.自己不愿出去 |
| B05 | 您的孩子一周上几节体育课？ | 1.低于三节,2.三节,3.四节,4.五节，5.大于五节 6.不知道 |
| B06 | 体育课一般在哪儿进行？ | 1.以室内为主 2.以室外（如操场等）为主（下雨才在室内） |

C、校外用眼情况

|  | 调查内容 | 选项 |
| --- | --- | --- |
| C01 | 在过去一周里，您的孩子平均每天放学后做作业或读书写字多长时间？ | 1.不到 1小时 2.1-2小时 3.2-3小时 4.3小时及以上 5.不知道 6.没有作业 |
| C02 | 在过去一周里，您的孩子参加英语、数学、写作等文化类补习班共多长时间？ | 1.不到 1小时 2.1-2小时 3.2-3小时 4.3小时及以上 5.不知道 6.无补习班（跳到C06） |
| C03 | 补习班采取的方式呢？ | 1.全部线上 2.全部线下 3.线上线下均有，以线上为主 4. 3.线上线下均有，以线下为主 |
| C04 | 您的孩子从几岁开始参加文化类补习班？ | 1.3岁以前 2.3岁 3.4岁 4.5岁 5.6岁及以后 |
| C05 | 为让孩子有更多时间做作业或上补习班,您会减少孩子运动的时间吗？ | 1.经常2.有时3.没有 |
| C06 | 您是否限制孩子看电视、玩电脑或电子游戏时间？ | 1.是 2.否 |

D、读写姿势

|  | 调查内容 | 选项 |
| --- | --- | --- |
| D01 | 您的孩子在读写时，胸口离桌子边沿超过一拳吗？ | 1.从不是 2.偶尔 3.经常 4.总是 |
| D02 | 您的孩子在读写时，眼睛距离书本超过一尺（ 33厘米，约 2个小号矿泉水瓶长）吗？ | 1.从不是 2.偶尔 3.经常 4.总是 |
| D03 | 您的孩子在读写时，手指距离笔尖一寸左右（ 3.3厘米）吗？ | 1.从不是 2.偶尔 3.经常 4.总是 |
| D04 | 老师或父母是否提醒您的孩子注意读写姿势？ | 1.从不 2.偶尔 3.经常 4.总是 |

E、电子屏幕使用情况

|  | 调查内容 | 选项 |
| --- | --- | --- |
| E01 | 在过去一周里，您的孩子平均每天因学习或非学习目的共用多长时间的电子屏幕产品（包括电视、电脑、手机、 ipad等电子产品等） | 1.我没有用过 2.不到 30分钟 3.30-60分钟 4.1-2小时 5.2-3小时 6.3小时及以上 |
| E02 | 您的孩子所在的班级老师上课时使用的多媒体类型是（可多选） | 1.电子白板2.投影幕布3.触摸电视4.不使用多媒体5.不知道 |
| E03 | 您的孩子所在的班级老师上课时平均每节课使用多媒体设备的时间是？ | 1.从来不使用 2.15分钟以内 3.15-30分钟 4.30分钟及以上 |

F、户外活动

|  | 调查内容 | 选项 |
| --- | --- | --- |
| F01 | 过去一周里，您的孩子平均每天在校外接触自然光的时间 | 1.不到 0.5小时 2.0.5-1小时 3.1小时及以上 |
| F02 | 过去一周里，您的孩子平均每天白天呆在户外的时间是多少（可累计）？ | 1.不到 1小时 2.1-2小时 3.2-3小时 4.3小时及以上 |
| F03 | 您和孩子知道足够长时间的户外活动对近视防控有非常好的作用吗？ | 1.不知道 2.印象不深刻 3.知道 |
| F04 | 如果专家告知您户外（如花园、阳台）做作业和看书对预防近视有非凡的意义，您的想法是？ | 1.今后完全可以考虑 2.无法实施 3.不予采纳 |

G、近视家族史

|  | 调查内容 | 选项 |
| --- | --- | --- |
| G01 | 孩子父母是否近视？ | 1.均不近视 2.一方近视 3.双方均近视 4.不知道 |
| G02 | 孩子父母是否高度近视？ | 1.均不近视 2.一方高度近视 3.双方均高度近视 4.不知道 |
| G03 | 孩子外公外婆是否近视？ | 1.均不近视 2.一方近视 3.双方均近视 4.不知道 |
| G04 | 孩子外公外婆是否高度近视？ | 1.均不近视 2.一方高度近视 3.双方均高度近视 4.不知道 |
| G05 | 孩子爷爷奶奶是否近视？ | 1.均不近视 2.一方近视 3.双方均近视 4.不知道 |
| G06 | 孩子爷爷奶奶是否高度近视？ | 1.均不近视 2.一方高度近视 3.双方均高度近视 4.不知道 |
