# Supplementary material for: High prevalence of myopia and low hyperopia reserve in 4411 Chinese primary school students and associated risk factors
Source: BMC Ophthalmol. 2022 May 11;22:212. doi: 10.1186/s12886-022-02436-5 (PMC9092685; doi:10.1186/s12886-022-02436-5)
Supplement: Supplementary file 3 — Additional file 3: Supplementary Material 3 (S3). Results of univariate analysis of the questionnaire for myopia and Influencing factors in Schoolchildren. [file 12886_2022_2436_MOESM3_ESM.doc]

Results of Univariate Analysis of the Questionnaire for Myopia and Influencing factors in Schoolchildren

A、Basic Information

|  | Questions | P value |
| --- | --- | --- |
| A01 | The educational attainment of your child's father | 0.758^a^ |
| A02 | The educational attainment of your child's mother | 0.410^a^ |
| A03 | Your child's weight at birth | 0.898^b^ |
| A04 | The way your child was born | 0.324^c^ |
| A05 | Whether your child is premature at birth (<37 weeks of gestation) | 0.237^d^ ,0.255^d^,0.660^d^  All >0.016667 |

B、Activities at School

|  | Questions | P value |
| --- | --- | --- |
| B01 | Were the desk and chair height adjusted according to your child's height? | 0.233^b^ |
| B02 | During the break between classes, does your child usually take outdoor activities? | 0.016^b^ |
| B03 | How many classes' recess in a day does your child have ten minutes to walk out of the classroom to go outdoors? | 0.182^c^ |
| B04 | What is the main reason why he or she can't go outdoors for ten minutes between classes? | 0.001^b^ |
| B05 | How many PE classes does your child have in a week? | 0.123^b^ |
| B06 | Where does PE class generally take place? | 0.780^c^ |

C、Activities after school

|  | Questions | P value |
| --- | --- | --- |
| C01 | In the past week, how long did your child do homework or read and write on average every day after school? | 0.076^c^ |
| C02 | In the past week, how long did your child take cram school classes such as English, math, and writing? | <0.001^b^ |
| C03 | Where did cram school classes take place? | 0.404^b^ |
| C04 | At what age does your child start to take cram school classes? | P=0,001^d^ between at 4 and after 6 years old,others were not significant |
| C05 | To give your children more time to do homework or go to cram school, will you reduce the time your child spends on exercise? | 0.087^b^ |
| C06 | Do you restrict your children's time to watch TV, play computer or video games? | 0.672^b^ |

D、Reading and writing posture

|  | Questions | P value |
| --- | --- | --- |
| D01 | When your child reads and writes, is his or her chest at proper distance (5-7cm) between the edge of the table? | 0.321^b^ |
| D02 | Are your child’s eyes approximately 33 cm away from the book when reading and writing? | 0.790^b^ |
| D03 | Does your child hold the pen about 3.3 cm away from the tip when reading and writing? | 0.797^b^ |
| D04 | Does the teacher or parent remind your child to pay attention to the reading and writing posture? | 0.901^c^ |

E、Electronic Screen Use

|  | Questions | P value |
| --- | --- | --- |
| E01 | In the past week, how long did your child use electronic products (including TVs, computers, mobile phones, ipads, etc.) each day for learning or other purposes on average? | 0.031^b^ |
| E02 | The type of multimedia used by the teacher in your child's class is (multiple choices available) | N/A |
| E03 | What is the average time that your child’s class teacher uses multimedia devices in each lesson? | 0.212^b^ |

F、Outdoor activities

|  | Questions | P value |
| --- | --- | --- |
| F01 | In the past week, the average time your child was exposed to natural light after school every day | 0.433^b^ |
| F02 | In the past week, how long did your child spend outdoors during the day on average? | 0.265^b^ |
| F03 | Are you and your child aware that outdoor activities have a good effect on myopia prevention and control? | 0.508^b^ |
| F04 | If an eye expert tells you that doing homework and reading outdoors (such as in the garden, on the balcony) is of great significance for preventing myopia, what would you do? | 0.953^c^ |

G、Family History of Myopia

|  | Questions | P value |
| --- | --- | --- |
| G01 | Do the child's parents have myopia? | 0.001^b^ |
| G02 | Do the child's parents have high myopia? | <0.001^c^ |
| G03 | Do the child's maternal grandparents have myopia? | 0.017^c^ |
| G04 | Do the child's maternal grandparents have high myopia? | 0.016^b^ |
| G05 | Do the child's paternal grandparents have myopia? | 0.002^c^ |
| G06 | Do the child's paternal grandparents have high myopia? | 0.013^c^ |

a: Mann-Whitney U test

b: Chi-square test

c: Fisher’s exact test

d: Fisher’s exact test, adjusted by Bonferroni’s method
